# Renal Replacement Therapy in Children in Lithuania: Challenges, Trends, and Outcomes

**DOI:** 10.3390/medicina54050078

**Published:** 2018-11-02

**Authors:** Ernestas Viršilas, Rimantė Čerkauskienė, Jūratė Masalskienė, Šarūnas Rudaitis, Diana Dobilienė, Augustina Jankauskienė

**Affiliations:** 1 Institute of Clinical Medicine, Vilnius University, LT-08406 Vilnius, Lithuania; rimante.cerkauskiene@gmail.com (R.Č.); augustinajankauskiene@yahoo.com (A.J.); 2 Department of Pediatrics, Medical Academy, Lithuanian University of Health Sciences, LT-50161 Kaunas, Lithuania; jurate.masalskiene@gmail.com (J.M.); rsarunas@gmail.com (Š.R.); dobdiana@yahoo.com (D.D.)

**Keywords:** epidemiology, end stage renal disease, renal replacement therapy, children, Lithuania

## Abstract

*Background and Objectives:* Pediatric renal replacement therapy (RRT) in Lithuania resumed in 1994 after a 12-year pause in renal transplantation. Management of end stage renal disease (ESRD) has changed, and outcomes have improved over decades. Our aim was to evaluate the dynamics of RRT in Lithuania in the period 1994–2015, describe its distinctive features, and compare our results with other countries. *Materials and Methods:* Data between 1994 and 2015 were collected from patients under the age of 18 years with ESRD receiving RRT. The data included: Hemodialysis (HD), peritoneal dialysis (PD), transplantation incidence and prevalence, transplant waiting time, dialysis modalities before transplantation, causes of ESRD and gender distribution in transplanted patients, and patient and graft survival. *Results:* RRT incidence and prevalence maintained an increase up until 2009. Sixty-four transplantations were performed. Juvenile nephronophthisis (25.9%) was the primary cause of ESRD in transplanted children. The transplant waiting time median was 8.0 months. The male to female ratio post-transplantation was 1.02. Patient survival after transplantation at 10 years was 90.0%, while graft survival for living (related) was 77.0% and 51.1% for deceased. Twelve patients died while on RRT. *Conclusions:* RRT numbers are increasing in Lithuania. HD is the primary treatment of choice before transplantation, with continued low numbers of preemptive transplantation. Patient survival post-transplantation is favorable, though graft survival is less satisfactory.

## 1. Introduction

Pediatric renal replacement therapy (RRT) in Lithuania resumed in 1994 after a 12-year pause, initially with hemodialysis (HD) for end stage renal disease (ESRD), then transplantation (Tx) in 1996, and the introduction of peritoneal dialysis (PD) in 1998. Management of ESRD has changed, with outcomes improving over the decades since [1]. Each method of RRT has its own advantages and disadvantages, and each is applied according to medical needs and institutional/national preferences [2,3,4]. According to the European Society of Pediatric Nephrology (ESPN)/European Renal Association-European Dialysis Transplantation Association (ERA-EDTA) Registry (2014), the treatment modality at RRT initiation was as follows: hemodialysis (HD)—41.2%; peritoneal dialysis (PD)—39.5%; pre-emptive transplantation—18.4% [5]. In Japan, PD predominated (61.7%), followed by pre-emptive transplantation (22.3%) and HD (16.0%) [6]. According to the United States’ renal data system, HD is its dominant modality with an increasing trend, while PD is on a steady decline [7]. As most African countries are unable to afford chronic RRT, there is lack of data and renal registries regarding the modality of choice from this region [8]. A study by Nakamura-Taira et al. [2] showed that professional care and dialysis-free days were considered advantages of HD, while independence, fewer hospital visits, and flexibility were considered benefits of PD. Sayin et al. [3] found no difference in the quality of life between HD, PD, and transplanted patients. Medical and technical aspects of the procedures dominated earlier clinical focus, while now the focus is on the psychosocial impact on the patient [3]. Our aim was to evaluate RRT dynamics in Lithuania over 22 years, describe its distinctive features, and compare our results with other countries.

## 2. Materials and Methods

All patients <16 years of age receiving RRT between 1 January 1994 and 31 December 2004 and <18 years between 1 January 2005 and 31 December 2015 were analyzed. Data was collected retrospectively using patient medical records from the only two pediatric nephrology centers in Lithuania (Vilnius, Kaunas) providing RRT for the pediatric population. The following parameters were analyzed: age; sex; incidence and prevalence of hemodialysis, peritoneal dialysis and renal transplantation (Tx); causes of ESRD in transplanted children; RRT before Tx; transplant waiting time; mean age at transplantation; sex distribution of transplanted children; patient and graft survival post-transplantation; and number and causes of death for patients requiring RRT. HD, PD, and Tx incidence were presented as absolute numbers per year, and period prevalence was expressed as the average per time period. Incidence and period prevalence were grouped into time periods to reflect dynamics. A five-year moving average was used to illustrate incidence trends. The incidence of children starting RRT was presented as the number of patients per million age-related population (pmarp). Tx waiting times were expressed as a median with interquartile range (IQR). RRT modality, causes of ESRD, and gender distribution were described in percentages. Patient and graft survival analysis was visualized using the Kaplan-Meier estimate. Statistical analysis was performed using SPSS 20.0 version (IBM Corp., Armonk, New York, NY, USA). We obtained a Regional Biomedical Research Ethics Committee approval (No. 158200-18/5-1039-535 and BE–2-62) to conduct this study.

## 3. Results

**Figure 1:**
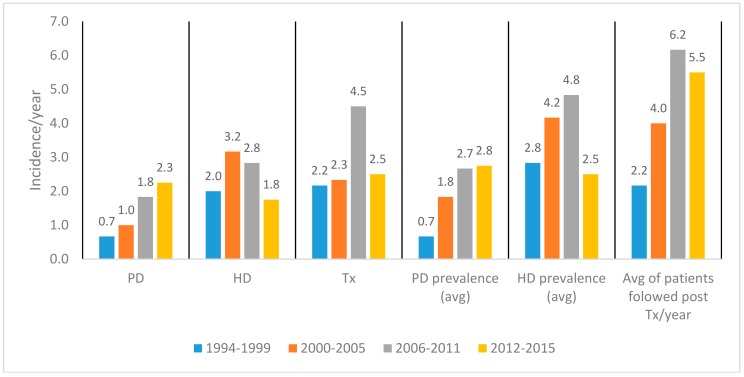
Peritoneal dialysis (PD), hemodialysis (HD), and transplantation (Tx) incidence per year and average (avg) period prevalence in Lithuania during 1994–2015.

**Figure 2:**
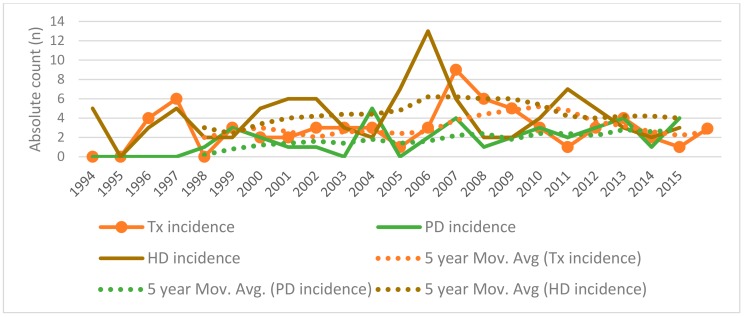
Peritoneal dialysis (PD), hemodialysis (HD), and transplantation (Tx) incidence with 5-year moving average trends in Lithuania during 1994—2015.

PD, HD, and kidney Tx incidence and period prevalence are presented in Figure 1. PD, HD, and Tx incidence trends are shown in Figure 2 with a five-year moving average calculated and visualized to reflect incidence dynamics. A total of 64 kidney transplantations were performed during the time period analyzed. Causes of ESRD in children were divided into three groups: glomerulonephritis and hemolytic uremic syndrome (HUS); hereditary nephropathies; and congenital anomalies of kidney and urinary tract (CAKUT). The dominant cause of ESRD was glomerulonephritis and HUS, followed by hereditary nephropathies; groups and causes of ESRD of transplanted patients are shown in Table 1. Juvenile nephronophthisis was the most frequent cause of ESRD before Tx. Distribution by sex post-Tx was nearly identical—50.7% were male and 49.3% female (M:F 1.02).

**Figure 3:**
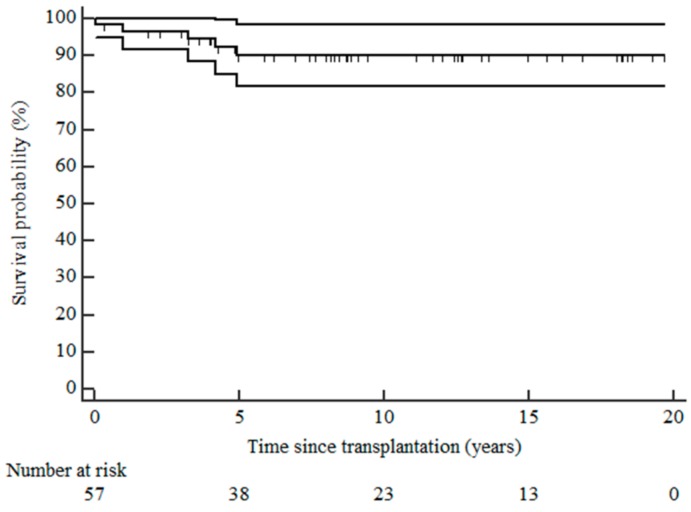
Kaplan-Meier curve for patient survival after transplantation with 95% confidence intervals.

**Figure 4:**
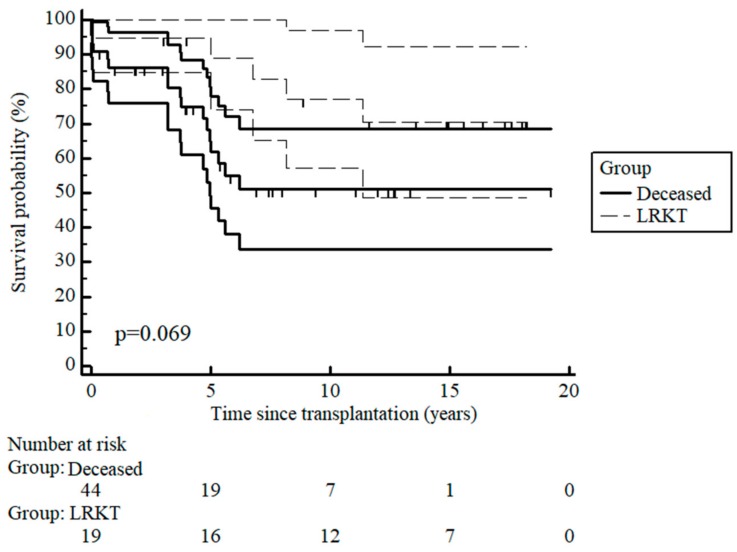
Kaplan-Meier curve for living-related kidney transplantation (LRKT) and cadaveric graft survival after transplantation with 95% confidence intervals.

During the 22-year study period, the most frequently used treatment modality was HD (72.7%), followed by PD (15.2%) and a combination of HD and PD (9.1%). Preemptive transplantation was only performed in 3.0% of patients. In the combination group, the change in dialysis was sequential for specific reasons: four patients transitioned from PD to HD before transplantation or as a temporary switch due to compromised peritoneum, and one shifted to PD as a preferred method after stabilization. The mean age at transplantation was 12.85 ± 3.28 years. The incident patients receiving different modalities of RRT age distribution were as follows: 18 in the 0–4 years age group, 16 in the 5–9 years age group, 24 in the 10–14 years age group, and 39 in the group over 14 years of age. The number of children starting RRT in relation to per million age-related population is presented in Table 2. The median transplant waiting time was 7.5 months (IQR 3—20.75), ranging from 0.66 months to 106 months. Patient survival rates after transplantation at 1, 5, and 10 years were 96.5%, 90.0%, and 90.0%, respectively (Figure 3). All living donations were from patient relatives. Survival rates for living-related kidney transplantation (LRKT) at 1, 5, and 10 years were 94.7%, 88.8%, and 77.0%, deceased graft survival rates were 86.2%, 61.7%, and 51.1%, respectively (Figure 4). Twelve patients died while receiving RRT due to sepsis (33.3%), stroke (25.0%), or other reasons (25.0%). Two patients (16.7%) died due to the inability to perform RRT for various reasons.

## 4. Discussion

The incidence of HD and PD showed a steady increase until 2007, after which HD rates stabilized and PD showed a tendency to increase. These findings are explained by the increased ESRD prevalence in our country, which might reflect better recognition and diagnostics of ESRD and the availability of the full range of RRT methods. Worldwide, the incidence of chronic kidney disease (CKD) and ESRD have not increased since 2010 [9]. The incidence of transplantation has varied greatly over the years. Kidney transplantation and RRT averages in Lithuania fall behind most Western European countries (Netherlands, UK, Portugal, Spain, France) [10,11]. It should be noted (Chesnaye at al. [11]) that the results are not completely comparable because of age limits for different study groups and follow-up periods. Renal transplantation as a treatment modality is most prominent in countries with a high gross domestic product (GDP) [10,12], but in Lithuania, the incidence of Tx is slightly higher than in other countries with a similar GDP [10]. Transplant waiting times also varied considerably within patients, but the majority received a transplant in less than 20 months, with decreasing waiting time as the years followed. This is similar to waiting times in other countries [10,13,14] and represents a favorable result, considering Lithuania’s GDP. It should also be noted that our country does not practice presumed consent for organ donation. Another peculiarity is our low rate of preemptive transplantation, which might reflect late referral to nephrologists and thus late recognition of ESRD [15]. Even though more positive outcomes are achieved from preemptive kidney Tx [16], it remains underutilized in Lithuania compared to other countries [10,17]. 

HD was the second most used dialysis modality in Lithuania after Tx. Two factors played a role: firstly, HD was introduced before PD for chronic patients; secondly, HD and PD are not covered equally by Lithuania’s healthcare system, in that PD materials are covered centrally by our Patients Fund, but the procedure itself is not on the approved list for reimbursement by the government, nor is payment for the relevant staff. This makes PD an unfavorable procedure for economic reasons, while the situation is just the opposite for HD. HD is fully funded, with HD centers widely spread throughout Lithuania and easily accessible for patients within a 50-km distance. Therefore, hospitals may be more inclined to perform HD rather than PD for economic reasons, despite its medical benefits [18], especially in younger children. 

Juvenile nephronophthisis was the most frequent disease causing ESRD before Tx, an unexplained peculiarity. Glomerulonephritis and HUS were the dominant causes of ESRD in our transplanted patients. Our results differ from other datasets, in which CAKUT was the leading cause of CKD [19,20] and ESRD [21,22] in transplanted children. Recent data from Lithuania shows a changing trend, with CAKUT becoming the more prominent cause of ESRD among children (unpublished data).

Although some European countries report that males receive renal transplantation more often [23] due to a male predisposition for ESRD, we did not find this tendency, as both sexes received equal numbers of transplants [23]. Considering that males are more predisposed to have CAKUT [24], our equal distribution amongst sexes could reflect inadequate recognition of the anomalies. 

The outcome of renal transplantation in children has improved over the last several decades [25,26]. The survival of living-related donor transplants was superior to deceased donor transplants for the first 5 years, which is similar to other reports [27,28,29], but this trend is lost by 10 years as also reported in other studies [26]. Patient survival rates after Tx were comparable to those of other countries [13,21]. Long-term outcome studies have shown that transplant survival is affected by many factors: the impact of donor source; recipient age; diagnoses; pre-operative immunotherapy and induction therapy; and early and late acute rejections in children that affect graft survival. These factors, however, were not within the scope of our study.

Twelve patients requiring RRT died. Of these 12, six were in dialysis and four died after Tx. Infection was the leading cause of death, followed by stroke. The literature demonstrates that patients receiving RRT are more prone to infections [30] due to immune dysregulation, immunosuppression (if Tx), and/or a constant site of infection (PD/HD). Stroke is one of the CKD-related cardiovascular complications and often is exacerbated by dialysis initiation and insufficient control of hypertension [31]. Our results demonstrated similar tendencies of mortality causes to those reported in other countries, with infection and cardiovascular complications being the leading causes of mortality [32,33]. Two deaths occurred due to an inability to perform RRT. PD could not be continued for a 6-month-old patient because of fungal peritonitis and compromised peritoneum. At that time, we did not have the technical ability to provide HD for infants. The second death was due to damaged vascular access and, subsequently, the inability to perform HD. PD was not an option for social reasons, as the parents refused to perform it at home. Today, both outcomes could be avoided, given improvements in hospital equipment and governmental social policies.

## 5. Conclusions

In conclusion, RRT has become increasingly prevalent in recent years. As the prevalence of PD has increased, so has the need for change in governmental policy to fund PD procedures. Transplant waiting times are improving and are satisfactory considering Lithuania’s GDP. Patient survival rates are favorable, but graft survival rates demand improvement. There were 12 deaths during the period analyzed, most of which occurred prior to 2004, with the majority influenced by infection and CVD complications.

## Figures and Tables

**Table 1:** Causes of end stage renal disease (ESRD) in transplanted children.

**GLOMERULONEPHRITIS AND HEMOLYTIC UREMIC SYNDROME**	**43.1%**
Acute tubular necrosis	3.4%
Chronic glomerulonephritis	6.9%
Denys-drash syndrome	3.4%
Diffuse proliferative glomerulonephritis	1.7%
Focal segmental glomerulosclerosis	8.6%
Frasier syndrome	3.4%
Goodpasture syndrome	3.4%
Hemolytic-uremic syndrome	5.2%
Membranoproliferative glomerulonephritis	3.4%
Rapidly progresive glomerulonephritis	3.4%
**HEREDITARY NEPHROPATHIES**	**39.7%**
Hereditary nephropathy (undifferentiated)	6.9%
Jeune syndrome	1.7%
Juvenile nephronophthisis	25.9%
Kidney polycystosis	5.2%
**CONGENITAL KIDNEY ANOMALIES**	**17.2%**
Bilateral kidney dysplasia	1.7%
Bronchio oto renal syndrome	1.7%
Hydronephrosis (congenital anomalies of kidney and urinary tract (CAKUT))	3.4%
Multicystic dysplastic kidney	1.7%
Reflux nephropathy	5.2%
Renal hypoplasia	3.4%

**Table 2:** Incidence of children starting renal replacement therapy (RRT) presented as per million age-related population (pmarp).

**Year**	**1994**	**1995**	**1996**	**1997**	**1998**	**1999**	**2000**	**2001**	**2002**	**2003**	**2004**
Tx preemptive pmarp	0	0	0	1.15	0	0	0	0	0	0	0
HD pmarp	4.36	0	3.39	3.45	2.35	2.39	4.88	5.01	9.12	4.08	2.84
PD pmarp	0	0	0	0	0	2.39	2.44	1.25	1.3	0	5.68
Population 0–16 years	916,489	899,771	883,993	868,060	851,968	836,823	820,267	797,861	767,595	736,176	703,879
**Year**	**2005**	**2006**	**2007**	**2008**	**2009**	**2010**	**2011**	**2012**	**2013**	**2014**	**2015**
Tx preemptive pmarp	0	0	0	0	0	0	0	0	0	0	1.91
HD pmarp	9.64	17.35	4.49	3.1	1.6	3.32	12.18	1.8	5.52	0	5.72
PD pmarp	0	2.89	4.49	1.55	3.2	1.66	3.48	3.59	3.68	0	3.81
Population 0–18 years	726,333	691,793	667,579	645,452	624,787	602,618	574,943	556,263	543,756	532,647	524,473
